# Local and systemic cytokine profiles in children with pneumonia-associated lung consolidation

**DOI:** 10.3389/fimmu.2025.1546730

**Published:** 2025-05-08

**Authors:** Jilei Lin, Jiande Chen, Shuhua Yuan, Mingyu Tang, Guijun Yang, Huishan Zhang, Wanlin Li, Hui Zhao, Jing Zhang, Lei Zhang, Yong Yin

**Affiliations:** ^1^ Department of Respiratory Medicine, Shanghai Children’s Medical Center, Shanghai Jiaotong University School of Medicine, Shanghai, China; ^2^ International Medical Department, Shanghai Children’s Medical Center, Shanghai Jiaotong University School of Medicine, Shanghai, China; ^3^ Department of Respiratory Medicine, Sanya Women and Children’s Hospital Affiliated to Hainan Medical College, Hainan Branch of Shanghai Children’s Medical Center, Sanya, Hainan, China; ^4^ Department of Respiratory Medicine, Linyi Maternal and Child Healthcare Hospital, Linyi, Shandong, China; ^5^ Shanghai Children’s Medical Center Pediatric Medical Complex (Pudong), Shanghai, China; ^6^ Pediatric Artificial Intelligence (AI) Clinical Application and Research Center, Shanghai Children’s Medical Center, Shanghai, China

**Keywords:** children, cytokine, pneumonia, lung consolidation, bronchoalveolar lavage fluid

## Abstract

**Objective:**

Lung consolidation (LC) in pediatric pneumonia could lead to complicated clinical outcomes, yet the underlying immunological mechanisms are not fully understood. This study aimed to investigate the roles of local and systemic cytokines in the development of pulmonary complications and disease progression in children with pneumonia-associated LC.

**Design:**

Conducted at the Shanghai Children’s Medical Center, this study included 169 children admitted between June 2022 and October 2023.

**Methods:**

We analyzed levels of fifteen cytokines in bronchoalveolar lavage fluid (BALF) and blood. Classification and regression tree (CART) analysis identified specific cytokines associated with pulmonary complications and hypoxemia.

**Results:**

In children with LC, most local cytokines were found at higher levels than systemic cytokines, with no apparent correlation between the two. Notably, an elevated level of IL-8 (≥ 6615 pg/ml) in BALF was associated with an increased risk of hypoxemia. Additionally, elevated levels of IL-4 and INF-γ in BALF were closely associated with the development of multi-segmental LC. Furthermore, elevated levels of IL-2R in BALF were significantly associated with the occurrence of atelectasis, in contrast to their levels in peripheral blood.

**Conclusion:**

IL-4, INF-γ, IL-2R, and IL-8 levels in BALF are closely associated with pulmonary complications and disease progression in children with LC. Exploring targeted immunomodulatory therapies in these children may mitigate lung injury caused by excessive local inflammatory responses.

## Introduction

Pneumonia remains one of the most prevalent childhood illnesses worldwide, making it a substantial contributor to pediatric hospitalization rates (1). It accounts for a significant portion of child mortality, particularly among those under the age of 5, with a heightened impact observed in developing regions (2). Lung consolidation (LC) refers to the pathological process in which the alveolar air content is replaced by fluid, inflammatory exudates, or cells, leading to a solidification-like appearance on radiological imaging. In children, LC can result in more severe clinical manifestations and complications such as prolonged hospital stays, increased risk of hypoxemia, and atelectasis. However, the mechanisms driving the varied outcomes associated with LC remain poorly understood (3).

During the course of pneumonia, previous studies have found that infections by different pathogens may lead to similar inflammatory responses (4, 5). Cytokines, as pivotal signaling molecules, assume a central role in the intricate orchestration of the immune response and the regulation of inflammatory processes (6). Therefore, cytokine profiles hold promise for unraveling the immune response mechanisms associated with pneumonia (7). The levels of cytokines in bronchoalveolar lavage fluid (BALF) is closely associated with pulmonary inflammation, elevated levels of interleukin(IL)-6 and tumor necrosis factor-alpha (TNF-α) have been documented as being closely associated with the severity of pneumonia (5). Therefore, analysis of cytokine profiles may reveal the underlying mechanisms responsible for the diverse clinical outcomes in children with pneumonia-associated LC, and provide therapeutic targets for lung injury caused by excessive inflammatory responses.

Considering the factors above, this study assessed the BALF and peripheral blood cytokine levels in children with pneumonia-associated LC. The aim of this study is to explore the differences in cytokine profiles between BALF and serum, and to elucidate the immune response mechanisms that influence outcomes of the pneumonia-associated LC.

## Methods

### Data source

This was a single-center, observational study conducted at the Shanghai Children’s Medical Center between June 2022 and October 2023. Data were collected from pediatric patients admitted to the Department of Respiratory Medicine. Ethical approval for the study was obtained from the Ethics Committee of the Shanghai Children’s Medical Center, and all procedures were conducted in accordance with the guidelines outlined in the Declaration of Helsinki. Patient information was managed with strict confidentiality and anonymity.

### Study population

Inclusion criteria for this study included: 1) children hospitalized due to pneumonia, 2) aged between 1 month and 18 years, 3) chest computed tomography (CT) scans showing lung consolidation affecting two or more segments, and 4) informed consent obtained from the guardians and from children aged over 8 years for participation in the study. Conversely, the exclusion criteria encompassed any of the following conditions: 1) patients whose guardians declined bronchoscopy examination and treatment, 2) patients with hematologic malignancies, immunodeficiency, tumors, congenital cardiopulmonary diseases, muscular dystrophy, or cystic fibrosis, 3) long term use of immunosuppressants, 4) situations where the guardian declined participation in the study, 5) cases with significant omissions of important information. A total of 257 children were initially enrolled. After screening by the exclusion criteria, 88 children were excluded, resulting in a final sample size of 169. The detailed information of screening process was shown in **Supplementary Figure S1**.

### Sample collection and cytokine testing

Samples (BALF and blood) were collected within 48 hours of admission to capture the acute immune response. We collected 2 milliliters (mL) of peripheral blood from children, placing the samples in EDTA-coated anticoagulant tubes. Plasma samples were obtained by centrifuging the blood at 3000g for 10 minutes and immediately stored at -80°C until further use. Five mL of BALF was collected during bronchoscopy at the site of the lesion selected based on pulmonary imaging. Final BALF samples were obtained by centrifuging the primary BALF at 4000g for 5 minutes and stored immediately at -80°C until further use.

We used a multiplex bead-based detection kit from Nanjing Atom Life Technology Co., LTD (https://mp.weixin.qq.com/s/4kmGjAWv0TN1gavD7RMp1g), which simultaneously measures 15 cytokines in a single assay. The kit was employed to assess the levels of 15 cytokines, including IL-1β, IL-2, soluble interleukin-2 receptor (IL-2R), IL-4, IL-5, IL-6, IL-8, IL-10, IL-12p70, IL-17, IL-18, Interferon alpha (IFN-α), IFN-γ, TNF-α, and tumor necrosis factor-beta (TNF-β). The reagent kit comprises 15 fluorescent coding microspheres, each respectively coupled with one of these 15 different monoclonal antibodies, mixed in a specific ratio. During the incubation process, the capture antibody microspheres capture the fifteen cytokines in the sample. Subsequently, a ‘capture antibody microsphere antigen biotin-labeled detection antibody SA-PE’ detection complex is formed after adding biotin-labeled paired detection antibodies and streptavidin phycoerythrin (SA-PE). The immunoassay was conducted using the Luminex MAGPIX detection system (MAGPIX, Luminex, USA). Standard curves and cytokine concentrations were determined using the Atansys software (Nanjing Atom Life Technology Co., LTD). Standardized methods for cytokine dosage in serum and BALF were referenced from previous studies (8). For cytokine values below the lower limit of detection (LLOD), the values were divided by the square root of 2 [LLOD/sqrt(2)].

### Data extraction

The collected data encompassed baseline information (sex, age, weight, height), clinical symptoms, chest imaging results, infectious pathogens, and diagnosis. This information was extracted from our PLRTI database (9). For the detection of infectious pathogens, different methods were employed based on the type of pathogen: 1) For viruses and Mycoplasma pneumoniae, qualitative polymerase chain reaction (PCR) was performed to identify the presence of these pathogens. Quantitative analysis was not conducted. 2) For bacteria, detection was performed using bacterial culture. The results were expressed as colony-forming units per milliliter (CFU/mL), which reflects the number of viable bacteria in the sample.

### Definitions

The diagnosis of pneumonia adhered to the 2019 edition of diagnostic and treatment standards for pneumonia in children in China (10). The definition of hypoxemia is as follows: 1) transcutaneous oxygen saturation ≤92% or arterial oxygen tension less than 60mmHg without supplemental oxygen; 2) increased respiratory rate; 3) presence of clinical signs such as nasal flaring and tracheal tug.

LC refers to the pathological process in which the alveolar air content is replaced by fluid, inflammatory exudates, or cells, leading to a solidification-like appearance on radiological imaging. Based on clinical observations that consolidation involving three or more segments often indicates more severe disease, we established the following operational definitions: consolidation confined to two segments was categorized as limited segmental lung consolidation (LSLC), while cases involving three or more segments were classified as multi-segmental lung consolidation (MSLC).

### Statistical analysis

Continuous variables were compared by using t-tests if variables were normally distributed and while were presented as X ± standard deviation (SD), while Wilcoxon test if variables were not normally distributed and were presented as medians with the 25th and 75th percentiles (P25, P75). Data normality was assessed using the Shapiro-Wilk test. Since most cytokines were not normally distributed, their levels were presented as medians with medians with the 25th and 75th percentiles (P25, P75). Categorical variables were analyzed by the χ2 test or the Fisher’s exact test and expressed as numbers (n) and percentages (%). Since most cytokines were not normally distributed, comparisons of cytokine levels were performed using non-parametric tests. Specifically, the Wilcoxon rank-sum test was used to compare cytokine levels between different patients, while the Wilcoxon signed-rank test was applied to compare cytokine levels in serum and BALF from the same patients. For the heatmap visualization of cytokines, Spearman’s rank correlation coefficients (r_s_) were calculated to assess monotonic relationships between cytokine levels in serum and BALF. The r_s_ values range from -1 to 1, where absolute values closer to 1 indicate stronger correlations and values near 0 suggest weaker associations. Corresponding p-values were computed for each pairwise comparison to determine statistical significance (p < 0.05).

To determine which cytokines were most predictive of pulmonary consolidation, we applied classification and regression tree (CART) analysis using the ‘rpart’ package in R software. CART, also known as recursive partitioning, constructs a decision tree by repeatedly splitting the data into increasingly homogeneous subsets based on the dependent variable (11). At each step, the algorithm selects the cytokine and its optimal cutoff value that yields the best separation between outcome groups. The resulting tree structure includes decision nodes that show the predicted class, the total number of observations in that node, and the class distribution (i.e., number of observations from each outcome group). The optimal tree was obtained by selecting the complexity parameter value that minimized the cross-validated error in the model’s complexity table, followed by pruning the initial tree using the prune() function. The final tree was visualized using the ‘rpart.plot’ package. P<0.05 was considered to indicate statistical significance. All statistical analyses were performed using R 4.1 software.

## Results

### Study population and the relationship between local and systemic cytokines

**Figure 1:**
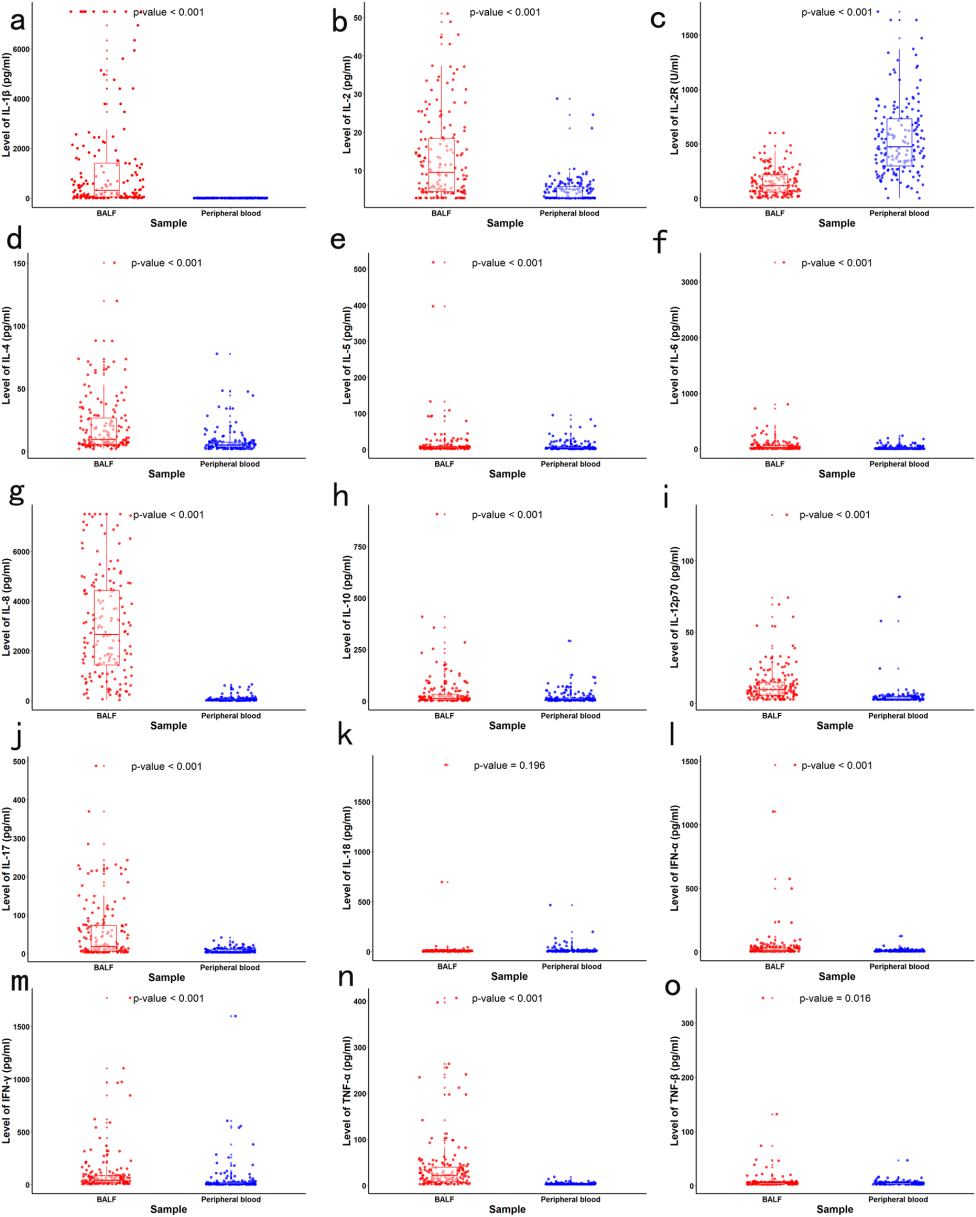
Concentrations of local (BALF) and systemic (peripheral blood) cytokines in children with LC. Paired comparisons between BALF and blood samples were conducted using the Wilcoxon signed-rank test, **(a)** IL-1β, **(b)** IL-2, **(c)** IL-2R, **(d)** IL-4, **(e)** IL-5, **(f)** IL-6, **(g)** IL-8, **(h)** IL-10, **(i)** IL-12p70, **(j)** IL-17, **(k)** IL-18, **(l)** IFN-α, **(m)** IFN-γ, **(n)** TNF-α, **(o)** TNF-β.

**Figure 2:**
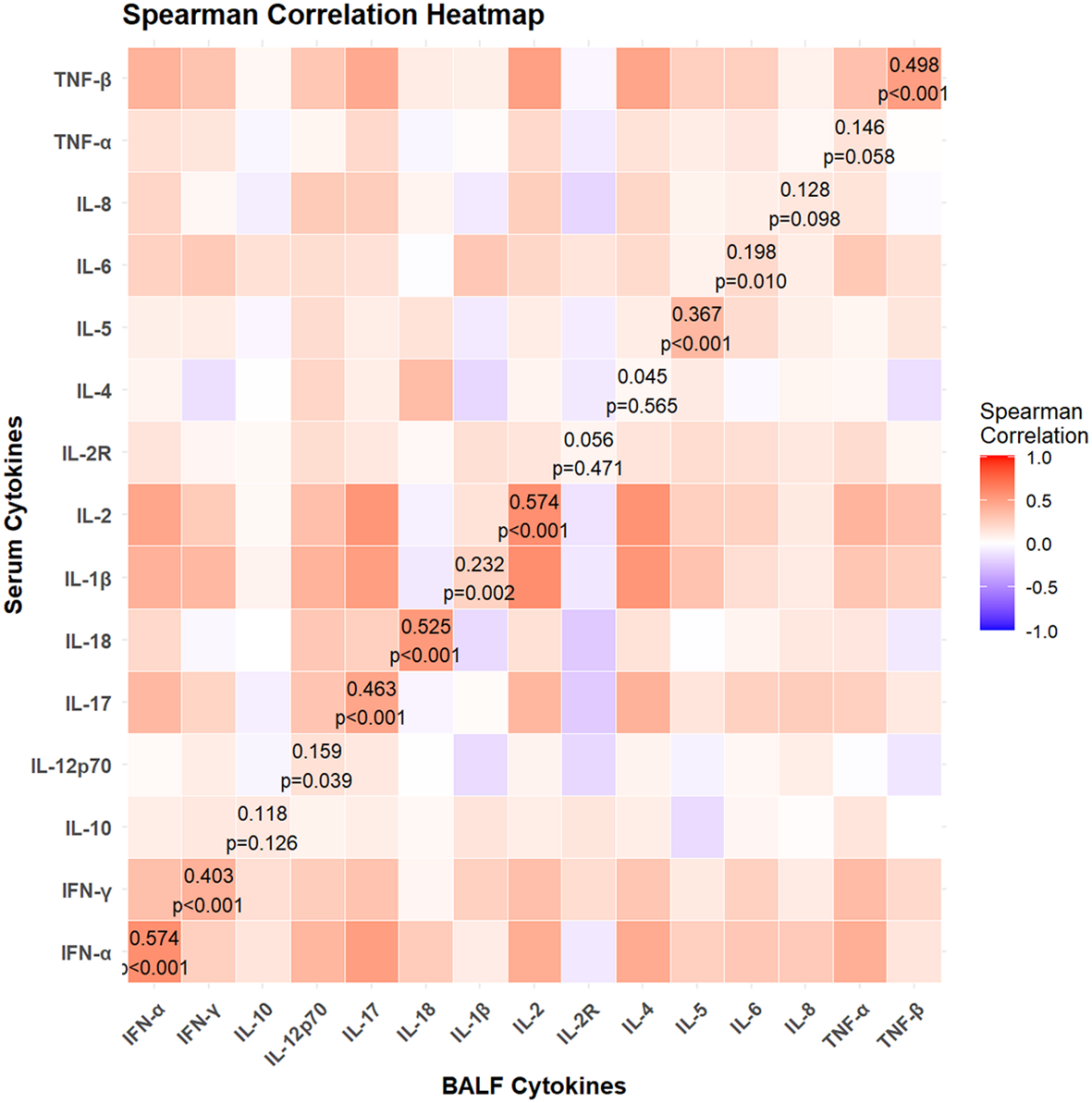
Spearman correlation heatmap between local and systemic cytokines in children with lung consolidation. The color intensity indicates the strength and direction of correlation (red for positive, blue for negative). Statistically significant correlations (p < 0.05) are labeled with corresponding correlation coefficients (r_s_) and p-values.

The mean age of the children included in our study was 83.47 ± 36.48 months old. **Supplementary Table S1** presents the baseline clinical characteristics of the study population. The concentrations of local and systemic cytokines were assessed. Significantly elevated levels of IL-1β, IL-2, IL-4, IL-5, IL-6, IL-8, IL-10, IL-12p70, IL-17, IFN-α, IFN-γ, TNF-α, and TNF-β were observed in BALF compared to peripheral blood (all p<0.05). In contrast, the concentration of IL-2R was significantly lower in BALF than in peripheral blood (p < 0.001). No significant difference was noted for IL-18 between the two sample types (p > 0.05) (**Figure 1**). The heatmap revealed varying degrees of association between local and systemic cytokines in children with pneumonia (**Figure 2**). Several cytokines showed moderate positive correlations, including IFN-α (r_s_ = 0.574), IL-18 (r_s_ = 0.525), and IL-2 (r_s_ = 0.574) (all p<0.001). In contrast, several cytokines exhibited little to no significant correlation, such as IL-10 (r_s_ = 0.118, p = 0.126), IL-4 (r_s_ = 0.045, p = 0.565), and IL-5 (r_s_ = 0.056, p = 0.471).

### Alterations in cytokine profiles in children with hypoxemia

Among these children, a total of 26 children had hypoxemia. No statistically significant differences were noted in the demographic characteristics between hypoxemia group and non-hypoxemia group (**Table 1**). Compared to children without hypoxemia, those with hypoxemia exhibited significantly elevated levels of IL-1β, IL-2, IL-4, IL-5, IL-6, IL-8, IL-10, IL-12p70, IL-17, IFN-α, IFN-γ, TNF-α, and TNF-β in BALF, as well as slightly higher IL-2 levels in blood (all p<0.05). No significant differences were observed in other cytokines in either BALF or blood.

**Table 1:** The demographic characteristics and cytokines between hypoxemia group and non-hypoxemia group.

Variable	Non-hypoxemia	Hypoxemia	P
	N=143	N=26	
Demographic characteristics			
Sex (boy, %)	59 (41.26%)	11 (42.31%)	>0.999
Age [Mean (SD), month old]	83.22 ± 34.20	84.81 ± 47.91	0.873
Height [Mean (SD), cm]	123.68 ± 22.41	122.00 ± 22.60	0.729
Weight [Mean (SD), kg]	26.38 ± 16.29	23.74 ± 12.15	0.343
Pathogens (Yes, %)			
Bacteria	18 (12.59%)	6 (23.08%)	0.270
Virus	24 (16.78%)	6 (23.08%)	0.622
MP	81 (56.64%)	15 (57.69%)	>0.999
Cytokines			
BALF			
IL-1β [Median (P25, P75), pg/ml]	202.10 (47.90, 927.75)	2009.05 (81.58, 5306.88)	0.005
IL-2 [Median (P25, P75), pg/ml]	8.80 (4.40, 16.65)	14.85 (7.88, 30.45)	0.013
IL-2R [Median (P25, P75), U/ml]	114.40 (51.95, 211.10)	134.00 (64.88, 308.20)	0.221
IL-4 [Median (P25, P75), pg/ml]	9.10 (5.40, 21.65)	37.25 (7.55, 68.08)	0.002
IL-5 [Median (P25, P75), pg/ml]	5.30 (3.70, 7.30)	10.65 (4.45, 25.10)	0.002
IL-6 [Median (P25, P75), pg/ml]	18.80 (8.30, 50.20)	73.35 (25.10, 152.28)	0.001
IL-8 [Median (P25, P75), pg/ml]	2580.70 (1392.45, 4249.60)	3978.70 (1464.10, 7305.23)	0.041
IL-10 [Median (P25, P75), pg/ml]	10.90 (5.20, 24.90)	25.55 (7.33, 87.88)	0.050
IL-12p70 [Median (P25, P75), pg/ml]	9.20 (5.70, 13.75)	15.70 (6.95, 27.60)	0.007
IL-17 [Median (P25, P75), pg/ml]	17.70 (6.30, 64.00)	95.30 (5.38, 221.30)	0.018
IL-18 [Median (P25, P75), pg/ml]	7.10 (4.35, 9.70)	8.10 (4.43, 10.73)	0.657
IFN-α [Median (P25, P75), pg/ml]	8.60 (3.54, 29.95)	40.60 (3.54, 51.75)	0.011
IFN-γ [Median (P25, P75), pg/ml]	38.80 (20.40, 75.75)	90.60 (20.83, 213.73)	0.028
TNF-α [Median (P25, P75), pg/ml]	19.40 (7.95, 34.40)	45.10 (8.78, 101.50)	0.030
TNF-β [Median (P25, P75), pg/ml]	5.50 (2.83, 6.30)	6.50 (3.20, 8.45)	0.029
Blood			
IL-1β [Median (P25, P75), pg/ml]	4.90 (3.25, 6.25)	5.35 (4.60, 6.18)	0.234
IL-2 [Median (P25, P75), pg/ml]	2.83 (2.83, 5.55)	5.00 (2.83, 6.40)	0.048
IL-2R [Median (P25, P75), U/ml]	451.50 (280.30, 713.15)	550.40 (458.23, 802.75)	0.070
IL-4 [Median (P25, P75), pg/ml]	4.60 (3.30, 6.90)	6.35 (4.18, 7.88)	0.112
IL-5 [Median (P25, P75), pg/ml]	4.40 (3.25, 6.75)	5.55 (3.80, 11.65)	0.078
IL-6 [Median (P25, P75), pg/ml]	8.30 (3.65, 27.35)	8.00 (4.23, 21.10)	0.946
IL-8 [Median (P25, P75), pg/ml]	33.60 (16.15, 63.70)	42.95 (23.08, 70.80)	0.328
IL-10 [Median (P25, P75), pg/ml]	7.80 (4.50, 14.60)	7.50 (4.85, 12.53)	0.722
IL-12p70 [Median (P25, P75), pg/ml]	4.00 (2.83, 4.85)	2.83 (2.83, 4.33)	0.084
IL-17 [Median (P25, P75), pg/ml]	5.40 (3.54, 11.55)	6.10 (3.54, 10.95)	0.924
IL-18 [Median (P25, P75), pg/ml]	7.70 (2.83, 10.25)	8.15 (6.75, 9.88)	0.305
IFN-α [Median (P25, P75), pg/ml]	6.90 (3.54, 10.45)	6.40 (3.54, 9.85)	0.374
IFN-γ [Median (P25, P75), pg/ml]	8.80 (3.54, 21.65)	8.65 (3.54, 12.63)	0.432
TNF-α [Median (P25, P75), pg/ml]	2.83 (2.83, 2.83)	2.83 (2.83, 2.83)	0.251
TNF-β [Median (P25, P75), pg/ml]	5.20 (2.83, 6.00)	5.50 (3.65, 6.80)	0.162

BALF, bronchoalveolar lavage fluid; MP, Mycoplasma pneumoniae.

**Figure 3:**
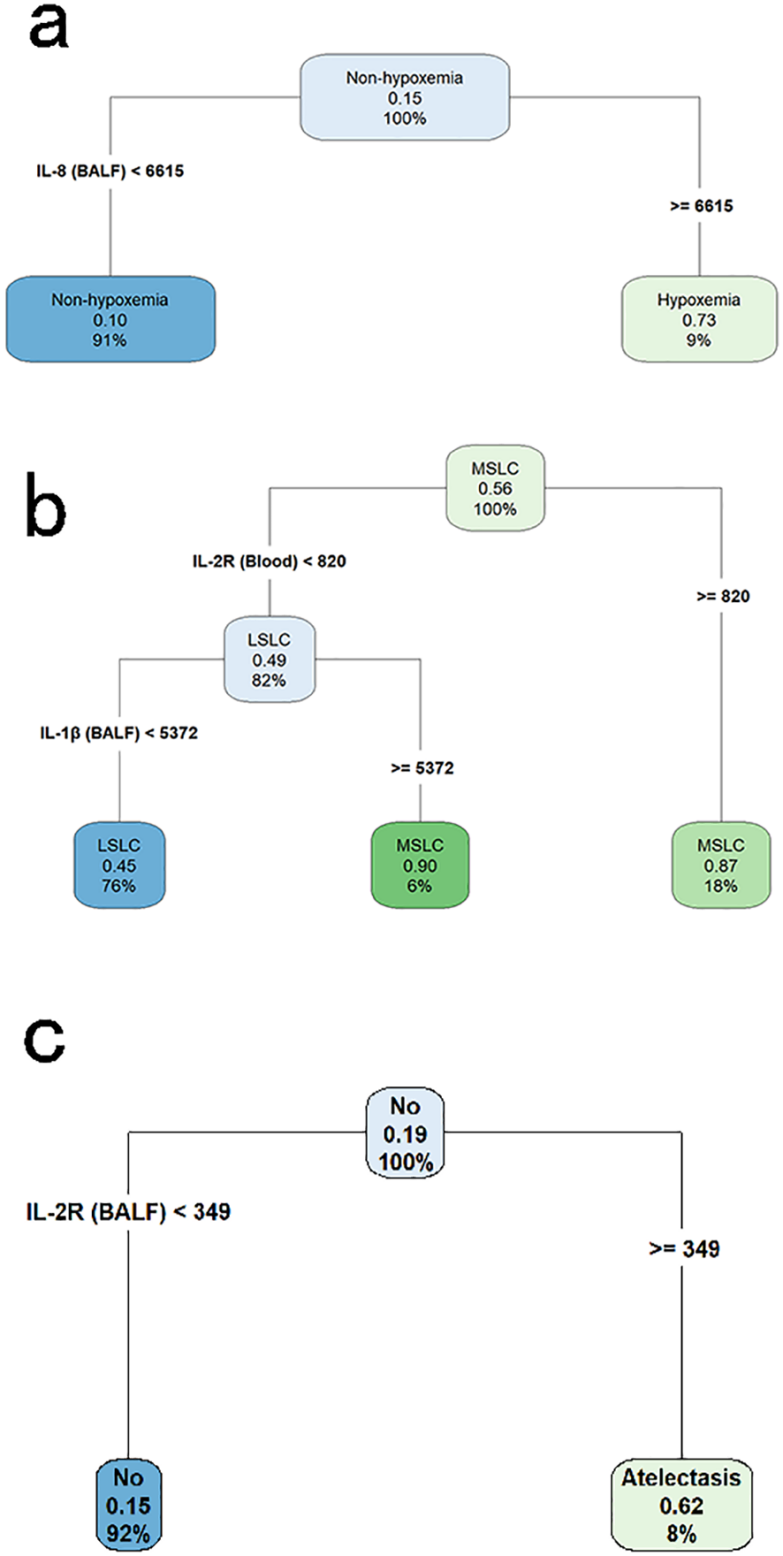
Classification and regression tree analyses for identifying key cytokine predictors of pulmonary complications in children with lung consolidation. **(a)** Decision tree for hypoxemia, with IL-8 in BALF identified as the primary discriminating cytokine. **(b)** Decision tree for multi-segmental lung consolidation, showing IL-2R in blood and IL-1β in BALF as key variables. **(c)** Decision tree for atelectasis, with IL-2R in BALF as the most informative predictor. Each node displays the predicted class, the probability of the outcome, and the percentage of samples represented.

We employed the CART algorithm for in-depth node classification of these children, focusing on the expression patterns of these significantly changed cytokines. The CART results indicated that elevated level of IL-8 (≥ 6615 pg/ml) in BALF was associated with an increased risk of hypoxemia (**Figure 3a**).

### Variations in cytokine profiles between LSLC and MSLC

Of the 169 children, 75 had LSLC and 94 had MSLC. Demographic characteristics were comparable between the LSLC and MSLC groups, with no significant differences in infectious pathogens (**Table 2**). Among these local and systemic cytokines, IL-1β, and TNF-α in BALF, as well as IL-2R in blood, were significantly elevated in the MSLC group compared with the LSLC group (all p< 0.05).

**Table 2:** the baseline and cytokines between different consolidation.

Variable	LSLC	MSLC	P
	N=75	N=94	
Demographic characteristics			
Sex (boy, %)	37 (49.33%)	33 (35.11%)	0.088
Age [Mean (SD), month old]	81.91 ± 36.91	84.71 ± 36.28	0.621
Height [Mean (SD), cm]	122.11 ± 24.24	124.47 ± 20.85	0.505
Weight [Mean (SD), kg]	24.85 ± 10.51	26.87 ± 18.88	0.381
Pathogens (Yes, %)			
Bacteria	13 (17.33%)	11 (11.70%)	0.412
Virus	11 (14.67%)	19 (20.21%)	0.462
MP	40 (53.33%)	56 (59.57%)	0.511
Intrapulmonary complications			
Pleural effusion	4 (5.33%)	42 (44.68%)	<0.001
NP	0 (0%)	4 (3.19%)	0.130
Plastic bronchus	0 (0%)	5 (5.32%)	0.116
Cytokines			
BALF			
IL-1β [Median (P25, P75), pg/ml]	121.60 (23.30, 699.80)	546.40 (68.10, 1901.95)	0.002
IL-2 [Median (P25, P75), pg/ml]	8.80 (4.30, 16.30)	10.05 (5.00, 19.80)	0.258
IL-2R [Median (P25, P75), U/ml]	92.30 (35.25, 178.85)	147.90 (64.50, 225.48)	0.053
IL-4 [Median (P25, P75), pg/ml]	8.30 (5.30, 28.10)	11.70 (6.03, 26.25)	0.208
IL-5 [Median (P25, P75), pg/ml]	5.40 (3.65, 7.30)	5.40 (3.95, 9.78)	0.436
IL-6 [Median (P25, P75), pg/ml]	21.60 (9.05, 52.95)	23.25 (8.78, 68.78)	0.314
IL-8 [Median (P25, P75), pg/ml]	2408.80 (989.90, 4305.85)	2,862.30 (1619.33, 4602.30)	0.134
IL-10 [Median (P25, P75), pg/ml]	9.40 (4.40, 35.80)	14.65 (5.60, 27.48)	0.144
IL-12p70 [Median (P25, P75), pg/ml]	9.90 (5.10, 14.20)	9.70 (6.00, 16.15)	0.516
IL-17 [Median (P25, P75), pg/ml]	17.80 (5.45, 71.55)	20.75 (7.20, 74.88)	0.263
IL-18 [Median (P25, P75), pg/ml]	8.20 (4.55, 9.90)	7.15 (4.20, 9.60)	0.369
IFN-α [Median (P25, P75), pg/ml]	13.30 (3.54, 33.25)	12.00 (3.54, 31.65)	0.930
IFN-γ [Median (P25, P75), pg/ml]	37.30 (13.20, 78.45)	46.05 (24.30, 88.60)	0.098
TNF-α [Median (P25, P75), pg/ml]	16.00 (6.65, 32.05)	25.05 (11.13, 47.70)	0.012
TNF-β [Median (P25, P75), pg/ml]	5.40 (2.83, 6.60)	5.55 (2.83, 6.50)	0.710
Blood			
IL-1β [Median (P25, P75), pg/ml]	4.90 (3.10, 6.00)	5.15 (3.60, 6.65)	0.203
IL-2 [Median (P25, P75), pg/ml]	2.83 (2.83, 5.20)	3.65 (2.83, 6.40)	0.263
IL-2R [Median (P25, P75), U/ml]	412.10 (265.10, 579.85)	585.25 (351.58, 839.75)	<0.001
IL-4 [Median (P25, P75), pg/ml]	5.60 (3.35, 8.15)	4.55 (3.43, 6.75)	0.326
IL-5 [Median (P25, P75), pg/ml]	4.40 (3.40, 7.30)	4.40 (3.13, 7.38)	0.678
IL-6 [Median (P25, P75), pg/ml]	8.30 (3.30, 22.55)	8.35 (4.10, 28.40)	0.291
IL-8 [Median (P25, P75), pg/ml]	35.70 (16.35, 58.70)	33.35 (18.00, 70.80)	0.722
IL-10 [Median (P25, P75), pg/ml]	6.20 (3.95, 12.05)	9.50 (5.15, 24.35)	0.006
IL-12p70 [Median (P25, P75), pg/ml]	2.83 (2.83, 4.75)	3.42 (2.83, 4.80)	0.966
IL-17 [Median (P25, P75), pg/ml]	5.60 (3.54, 11.65)	5.45 (3.54, 11.50)	0.835
IL-18 [Median (P25, P75), pg/ml]	8.00 (4.20, 10.35)	7.35 (2.83, 10.18)	0.375
IFN-α [Median (P25, P75), pg/ml]	7.60 (3.54, 12.75)	6.55 (3.54, 9.85)	0.179
IFN-γ [Median (P25, P75), pg/ml]	8.50 (3.54, 19.55)	9.50 (5.08, 21.68)	0.291
TNF-α [Median (P25, P75), pg/ml]	2.83 (2.83, 2.83)	2.83 (2.83, 4.15)	0.120
TNF-β [Median (P25, P75), pg/ml]	5.10 (2.83, 6.00)	5.40 (2.83, 6.10)	0.341

LSLC, limited segmental lung consolidation; MSLC,multi-segmental lung consolidation; NP, necrotizing pneumonia.

The CART results revealed a pivotal node at the top level of the tree, indicating that children with IL-2R levels ≥ 820 U/ml in blood were at a higher risk of MSLC. Among those with lower IL-2R levels, further stratification was based on IL-1β concentrations in BALF, where higher IL-1β levels (≥ 5372 pg/ml) were also associated with MSLC (**Figure 3b**).

### Changes in cytokine profiles in children with atelectasis

Among children with LC, a total of 32 children had atelectasis. No statistically significant differences in demographic characteristics were observed between the atelectasis group and the non-atelectasis group (**Table 3**). Notably, levels of IL-1β, IL-2R, and IL-12p70 in BALF, as well as IL-2R and IL-10 in blood, were significantly higher in children with atelectasis compared to those without. These elevated cytokine levels were utilized in constructing the CART, where a pivotal node at the top level of the tree indicated a higher risk of atelectasis in children with IL-2R levels ≥349 U/ml in BALF (**Figure 3c**).

**Table 3:** The demographic characteristics and cytokines between atelectasis group and non-atelectasis group.

Variable	Non-atelectasis	Atelectasis	P
	N=137	N=32	
Demographic characteristics			
Sex (boy, %)	60 (43.80%)	10 (31.25%)	0.272
Age [Mean (SD), month old]	82.98 ± 36.29	85.56 ± 37.77	0.727
Height [Mean (SD), cm]	123.28 ± 22.98	124.03 ± 19.89	0.852
Weight [Mean (SD), kg]	26.26 ± 16.62	24.73 ± 11.20	0.530
Pathogens (n, %)			
Bacteria	22 (16.06%)	2 (6.25%)	0.250
Virus	24 (17.52%)	6 (18.75%)	>0.999
MP	78 (56.93%)	18 (56.25%)	>0.999
Cytokines			
BALF			
IL-1β [Median (P25, P75), pg/ml]	193.50 (36.60, 1,033.80)	694.00 (151.70, 1,795.10)	0.013
IL-2 [Median (P25, P75), pg/ml]	9.10 (4.40, 17.60)	12.05 (6.08, 25.58)	0.063
IL-2R [Median (P25, P75), U/ml]	108.20 (42.40, 199.40)	197.90 (69.25, 298.90)	0.003
IL-4 [Median (P25, P75), pg/ml]	9.10 (5.40, 26.60)	14.05 (6.90, 24.70)	0.230
IL-5 [Median (P25, P75), pg/ml]	5.40 (3.90, 8.00)	5.10 (3.85, 8.50)	0.763
IL-6 [Median (P25, P75), pg/ml]	22.10 (8.20, 66.20)	22.65 (10.95, 60.63)	0.810
IL-8 [Median (P25, P75), pg/ml]	2,682.20 (1,160.30, 4,409.50)	2,605.00 (1,760.23, 4,800.28)	0.261
IL-10 [Median (P25, P75), pg/ml]	11.10 (5.10, 32.60)	14.55 (6.35, 28.88)	0.434
IL-12p70 [Median (P25, P75), pg/ml]	9.20 (5.50, 14.10)	11.65 (6.78, 26.68)	0.037
IL-17 [Median (P25, P75), pg/ml]	18.30 (6.10, 71.80)	30.60 (6.28, 99.05)	0.470
IL-18 [Median (P25, P75), pg/ml]	7.00 (4.20, 9.70)	9.25 (5.13, 10.68)	0.090
IFN-α [Median (P25, P75), pg/ml]	14.00 (3.54, 32.40)	10.05 (3.54, 33.43)	0.980
IFN-γ [Median (P25, P75), pg/ml]	40.40 (19.80, 85.60)	45.05 (24.53, 100.30)	0.338
TNF-α [Median (P25, P75), pg/ml]	20.10 (7.60, 37.40)	25.40 (12.88, 54.45)	0.088
TNF-β [Median (P25, P75), pg/ml]	5.50 (2.83, 6.50)	5.50 (2.83, 6.53)	0.613
Blood			
IL-1β [Median (P25, P75), pg/ml]	5.00 (3.40, 6.20)	5.00 (2.86, 6.25)	0.855
IL-2 [Median (P25, P75), pg/ml]	2.83 (2.83, 5.60)	4.35 (2.83, 6.40)	0.383
IL-2R [Median (P25, P75), U/ml]	443.40 (284.40, 671.90)	670.70 (453.20, 909.60)	0.002
IL-4 [Median (P25, P75), pg/ml]	4.80 (3.30, 7.30)	5.95 (4.00, 7.88)	0.132
IL-5 [Median (P25, P75), pg/ml]	4.40 (3.40, 7.50)	4.20 (2.12, 6.65)	0.276
IL-6 [Median (P25, P75), pg/ml]	8.00 (3.60, 24.30)	11.05 (4.83, 38.65)	0.218
IL-8 [Median (P25, P75), pg/ml]	33.60 (15.60, 67.30)	41.65 (22.83, 74.98)	0.187
IL-10 [Median (P25, P75), pg/ml]	7.20 (4.30, 12.90)	12.85 (6.95, 27.48)	0.013
IL-12p70 [Median (P25, P75), pg/ml]	2.83 (2.83, 4.70)	2.83 (2.83, 4.93)	0.884
IL-17 [Median (P25, P75), pg/ml]	5.40 (3.54, 11.00)	6.20 (3.54, 14.45)	0.627
IL-18 [Median (P25, P75), pg/ml]	7.80 (2.83, 9.70)	8.60 (6.85, 11.40)	0.057
IFN-α [Median (P25, P75), pg/ml]	6.90 (3.54, 10.20)	6.80 (3.54, 9.95)	0.591
IFN-γ [Median (P25, P75), pg/ml]	8.50 (3.54, 20.40)	10.75 (5.93, 23.00)	0.225
TNF-α [Median (P25, P75), pg/ml]	2.83 (2.83, 2.83)	2.83 (2.83, 4.20)	0.378
TNF-β [Median (P25, P75), pg/ml]	5.20 (2.83, 6.00)	5.45 (4.40, 6.10)	0.561

BALF, bronchoalveolar lavage fluid; MP, Mycoplasma pneumoniae.

## Discussion

In this study, we explored the intricate landscape of local and systemic cytokine expression in children with LC. Noteworthy findings include significant differences in cytokine profiles between BALF and blood, with elevated levels of key inflammatory markers like IL-1β, IL-2, IL-4, IL-5, IL-6, IL-8, IL-10, IL-12p70, IL-17, IFN-α, IFN-γ, TNF-α, and TNF-β in BALF indicating a localized inflammatory response in children with LC. IL-1β, IL-2R, and IL-8 are closely associated with pulmonary complications and disease progression in pediatric pneumonia with LC.

The alignment of local and systemic immune responses remains inconsistent in respiratory infections (12, 13). BALF offers a more direct reflection of immune activity at the infection site than peripheral blood, which may provide a diluted representation of the local response (14). Our correlation analysis confirmed that some cytokines exhibit parallel changes (e.g., IL-2, IFN-α, IL-18), whereas others differ substantially. This underscores the importance of assessing the local cytokine environment for a more precise understanding of pathological mechanisms, particularly in severe pediatric pneumonia. Currently, there is no consensus regarding the role cytokines play in the severity of pneumonia (15, 16). Luo’s research demonstrated that circulating levels of IL-4 and IFN-γ are not associated with the severity of COVID-19 symptoms (17). However, our study suggests that elevated levels of IL-4 and INF-γ in BALF are closely associated with the development of MSLC, which often represents more severe pneumonia in children. These disparate results suggest that systemic cytokine levels cannot be equated with those in BALF, highlighting the latter’s more direct involvement in the pathogenesis of pneumonia. Therefore, our study, through a comprehensive analysis of cytokines in peripheral blood and BALF, provides deeper insights into the potential mechanisms underlying the progression and severity of lung pathology in pneumonia.

In clinical practice, children with pneumonia accompanied by LC often do not exhibit hypoxemia (18). CART analysis revealed that heightened local levels of IL-8 in BALF may impair oxygen exchange in the pulmonary alveoli in LC. Previous studies have found that IL-8 is closely associated with the development of ARDS, which may explain some of our results (19, 20). Therefore, in children with LC and hypoxemia, targeting IL-8 for therapeutic modulation may be a potential focus of future research, with the aim of reducing mortality in pediatric pneumonia.

Interestingly, elevated IL-2R levels in BALF are significantly associated with the occurrence of atelectasis, rather than in peripheral blood. It is well known that elevated IL-2R levels in BALF are associated with increased T cell activation and immune system activation (21, 22). Notably, peripheral blood IL-2R was also significantly elevated in children with MSLC. This finding suggests that systemic IL-2R elevation might be used as a prognostic indicator or a marker of potential complications. Previous studies have primarily focused on systemic IL-2R levels as a marker of systemic inflammatory responses after infection, even suggesting a link to hemophagocytic syndromes (23, 24). In contrast, our results indicate that local IL-2R levels in BALF are more closely associated with pulmonary complications, while systemic IL-2R levels may reflect the overall burden of disease severity in MSLC. It remains unclear whether IL-2R serves as a biomarker indicating the intensity of the immune response or can be targeted therapeutically to reduce the occurrence of atelectasis or other complications. Therefore, further analysis is needed to explore the role of the IL-2/IL-2R pathway in different contexts of pneumonia.

There were several strengths in this study. Firstly, the research specifically targeted the pediatric population, addressing a critical gap in the literature. Pediatric pneumonia has unique characteristics, and this study contributes valuable insights to this domain. Secondly, the study not only examines cytokine levels but also correlates them with clinical outcomes such as hypoxemia and complications like atelectasis. This connection between cytokines and real-world clinical consequences enhances the practical applicability of the research. Finally, by identifying specific cytokine profiles associated with disease severity and complications, the research lays the groundwork for personalized medicine approaches. Tailoring interventions based on individual immune responses, particularly in children with pneumonia who exhibit excessive immune and inflammatory reactions, may be a promising avenue for future research.

Limitations of this study should also be noted. Firstly, Firstly, it is noteworthy that factors such as previous or concurrent immunomodulant therapies (e.g., macrolides, corticosteroids, high-dose IVIG), environmental exposures (e.g., passive smoking, air pollution), prematurity, vaccination status, and history of respiratory conditions (e.g., asthma, RSV infections) could influence cytokine levels. These variables represent important potential confounders that may bias the observed cytokine profiles. Our current data did not allow for in-depth subgroup analyses to fully account for their effects, but future multicenter studies with larger cohorts and more comprehensive data collection may address these limitations. Secondly, although all samples were collected early during hospitalization, and the majority of patients received cephalosporins or penicillin-class antibiotics at admission, the potential influence of antibiotic use on serum cytokines cannot be entirely ruled out and warrants further investigation. Thirdly, the study focused on a specific panel of cytokines, and other potentially relevant cytokines or inflammatory markers may not have been included. A broader cytokine profile analysis could provide a more comprehensive view of the immune response. Another limitation of this study is the lack of long-term follow-up data on serum cytokine levels. While we observed significant differences in serum cytokines during the acute phase of pneumonia, it remains unclear whether these changes persist or normalize over time. Future studies with longitudinal designs could collect serum samples at 6 and 12 months post-recovery to assess whether the observed cytokine alterations are transient or indicative of long-term immune dysregulation.

In addition to the cytokine profiles identified in this study, future research should explore the potential correlations between cytokine patterns and environmental factors such as pollution exposure, as well as their interactions with respiratory and gut dysbiosis. Air pollution disrupts immune responses and exacerbates inflammation, potentially altering cytokines like IL-8 and TNF-α (25–27). Gut microbiota, via the “gut-lung axis,” regulates systemic immunity, and dysbiosis may increase infection susceptibility. Investigating these interactions could reveal novel therapeutic targets for mitigating lung injury in children.

## Conclusion

In children with pneumonia-associated LC, local cytokine levels are significantly higher than systemic cytokine levels, and systemic cytokine levels cannot directly reflect the levels of local cytokines. IL-1β, IL-2R, and IL-8 are closely associated with pulmonary complications and disease progression in pediatric pneumonia with LC. Future research targeting these cytokines and their related pathways may play an important role in developing specific immunomodulatory therapies to reduce lung injury caused by excessive inflammatory responses in children with pneumonia.

## Data Availability

The raw data supporting the conclusions of this article will be made available by the authors, without undue reservation.
