# Supplementary material for: Local and systemic cytokine profiles in children with pneumonia-associated lung consolidation
Source: Front Immunol. 2025 May 8;16:1546730. doi: 10.3389/fimmu.2025.1546730 (PMC12094972; doi:10.3389/fimmu.2025.1546730)
Supplement: Supplementary file 1 [file DataSheet1.pdf]

Table S1: The clinical characteristics of included children

	Pneumonia N=169
**Demographic characteristics**	
Sex (boy, %)	70 (41.42%)
Age [Mean (SD), month old]	83.47±36.48
Height [Mean (SD), cm]	123.42±22.38
Weight [Mean (SD), kg]	25.97±15.72
**Clinical features**	
Fever (Yes, %)	157 (92.90%)
Cough (Yes, %)	
No	1 (0.59%)
Daytime	22 (13.02%)
Night	58 (34.32%)
Daytime and night	22 (13.02%)
Without a clear time pattern	66 (39.05%)
Expectoration (Yes, %)	161 (95.27%)
Wheeze (Yes, %)	19 (11.24%)
**Physical examination**	
Crackles (Yes, %)	82 (48.52%)
Wheezes (Yes, %)	16 (9.47%)

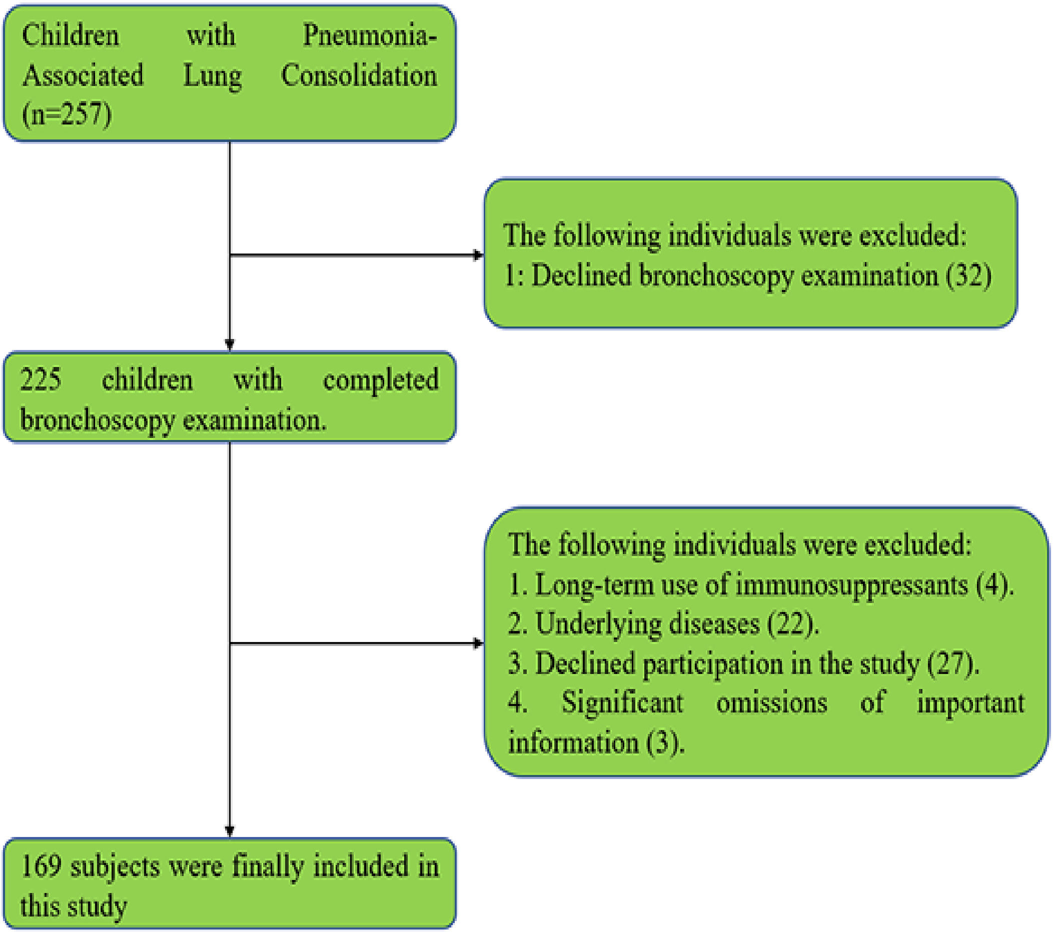

Figure S1: Flow diagram of the selected cases.
